# Impact of Rootstocks and Training Systems on Secondary Metabolites in the Skins and Pulp of *Vitis labrusca* and Brazilian Hybrid Grapes

**DOI:** 10.3390/plants14121766

**Published:** 2025-06-10

**Authors:** Francisco José Domingues Neto, Marco Antonio Tecchio, Silvia Regina Cunha, Harleson Sidney Almeida Monteiro, Ricardo Figueira, Aline Nunes, João Domingos Rodrigues, Elizabeth Orika Ono, Mara Fernandes Moura-Furlan, Giuseppina Pace Pereira Lima

**Affiliations:** 1 Sao Paulo State University (UNESP), School of Agricultural and Veterinary Sciences, Jaboticabal 14884-900, SP, Brazil; 2 Sao Paulo State University (UNESP), School of Agricultural Sciences, Botucatu 18618-689, SP, Brazil; marco.a.tecchio@unesp.br (M.A.T.); silvia.agro@mxb.com.br (S.R.C.); harleson.sa.monteiro@unesp.br (H.S.A.M.); ricardofigueira@hotmail.com (R.F.); 3 Sao Paulo State University (UNESP), Institute of Biosciences, Botucatu 18618-689, SP, Brazil; alinenunes_bio@hotmail.com (A.N.); joao.domingos@unesp.br (J.D.R.); elizabeth.o.ono@unesp.br (E.O.O.); finalima@gmail.com (G.P.P.L.); 4 Agronomic Institute of Campinas (IAC), Jundiaí 13214-820, SP, Brazil; mouram@iac.sp.gov.br

**Keywords:** phenolic compounds, vertical shoot position, antioxidant capacity

## Abstract

Grapes are rich in bioactive compounds, including phenolics and anthocyanins, which exhibit antioxidant properties and offer potential health benefits. The accumulation of these compounds is influenced by agronomic practices, particularly rootstock selection and training systems. This study evaluated the effects of different rootstocks (‘IAC 766 Campinas’ and ‘106-8 Mgt’) and training systems (low and high vertical shoot positioning) on the polyphenolic composition and antioxidant activity in the skins and pulps of *Vitis labrusca* and Brazilian hybrid grapes. The analyses included total phenolics, total flavonoids, monomeric anthocyanins, and antioxidant activity (DPPH and FRAP assays), as well as the individual polyphenolic profile in grape skins. The results indicated that both rootstock and training system significantly affected the accumulation of bioactive compounds and antioxidant capacity. Grapes trained on high trellises exhibited higher concentrations of bioactive compounds, while those from low trellises showed an enhanced phenolic composition. Among *Vitis labrusca* varieties, ‘Bordô’ had the highest bioactive compounds, while ‘Isabel’ stood out for specific phenolic acids. In hybrid cultivars, the ‘106-8 Mgt’ rootstock boosted antioxidant compounds, while ‘IAC 766 Campinas’ enhanced flavonoid, anthocyanin, and phenolic acid levels. Malvidin-3-O-glucoside emerged as the predominant anthocyanin. These findings underscore the importance of optimizing rootstock selection and training systems to enhance the phenolic composition and antioxidant potential of grapes.

## 1. Introduction

Grapes are recognized as an important source of phenolic compounds, which possess significant biological properties, particularly antioxidant compounds [1]. Phenolic compounds, especially flavonoids and anthocyanins, are critical determinants of the antioxidant activity of fruits and vegetables. They play a fundamental role in human nutrition and are associated with preventing cardiovascular diseases, cancer, and neurodegenerative disorders [2]. Beyond their nutritional benefits, these compounds also contribute to the commercial quality of red grapes. Anthocyanins, in particular, play a crucial role in determining the color and appearance of the berries, making them important phytochemicals for the economic value of grapes [3].

Given the increasing demand for products with high antioxidant capacity, research focused on diversifying viticultural production is warranted. The wide genetic variability among cultivars results in grapes with distinct flavor and color profiles, which are directly associated with the content and composition of polyphenols [2,3]. In addition to cultivar selection, the choice of rootstocks and training systems can significantly influence grape quality by modifying the accumulation of bioactive compounds, particularly phenolics and anthocyanins, as well as antioxidant activity. Both rootstock and training system directly affect the physicochemical parameters of the berries, such as pH, titratable acidity, and soluble solids content, all of which are related to fruit quality [4,5]. Studies demonstrate that rootstock selection influences the composition of both grapes and must [5,6,7,8]. However, there remains a gap in research evaluating the effects of training systems on the accumulation of bioactive compounds in grapes.

In *Vitis labrusca* and hybrid grapes, agronomic factors such as rootstock selection and training systems may be even more relevant due to their distinct metabolic profile when compared to *Vitis vinifera*. These grape species naturally exhibit higher levels of certain phenolic compounds, particularly anthocyanins and flavonols, which contribute not only to color stability but also to the sensory characteristics of the fruit and derived products [9]. Understanding how these metabolites are influenced by vineyard management practices is crucial for optimizing production aimed at both the fresh market and processed products, ensuring high nutritional and commercial value.

Moreover, the effects of environmental conditions, such as temperature, solar radiation, and water availability, interact with the choice of rootstocks and training systems, further shaping the chemical and biochemical profile of grapes [10]. Some training systems enhance light interception and airflow, which can lead to increased anthocyanin synthesis, while others may reduce excessive radiation exposure that could cause phenolic degradation [5]. Similarly, rootstocks affect water and nutrient uptake efficiency, potentially altering stress responses and, consequently, secondary metabolism [11]. Despite their importance, studies addressing these combined factors in *Vitis labrusca* and hybrid grapes remain scarce, highlighting the need for further investigation.

Considering that various factors regulate the concentration of bioactive compounds in grapes, this study aimed to evaluate the influence of rootstocks and training systems on the accumulation of secondary metabolites, including phenolics, flavonoids, and anthocyanins, as well as on the antioxidant activity of the skins and pulp of *Vitis labrusca* and hybrid grapes.

## 2. Materials and Methods

### 2.1. Experimental Design and Treatments

The experiment was conducted using a completely randomized design in a 2 × 2 factorial scheme, consisting of two rootstocks (‘IAC 766 Campinas’ (‘106-8 Mgt’ × *Vitis caribaea*) and ‘106-8 Mgt’ (*Vitis riparia* × (*Vitis cordifolia* × *Vitis rupestris*))) and two training systems. The Low Trellis system had three wires positioned at 1.0, 1.3, and 1.6 m, while the High Trellis system had four wires positioned at 1.0, 1.3, 1.6, and 2.0 m, allowing for greater canopy volume. The treatments were evaluated in four grape varieties: ‘Bordô’ and ‘Isabel’ (*Vitis labrusca*), and the hybrids ‘IAC 138-22 Máximo’ (Seibel 11342 × Syrah) and ‘BRS Violeta’ (‘Niagara Rosada’ × ‘Bordô’), with three replicates per treatment.

### 2.2. Sample Collection and Preparation

Grapes were harvested from an experimental area in Jundiaí, São Paulo (23°06′ S, 46°55′ W, 745 m elevation). From each plot, 200 berries were randomly collected from different positions within the clusters, including both the apical and basal portions, to capture the variability within each treatment. The berries were then separated into skins and pulps, frozen in liquid nitrogen, and stored at −80 °C until analysis. The samples were ground in a porcelain mortar and analyzed in triplicate.

### 2.3. Biochemical Analyses of Grape Skins and Pulps

The contents of total phenolic compounds (TPC), total flavonoids (TF), total monomeric anthocyanins (TMA), and antioxidant activity via 2,2-diphenyl-1-picrylhydrazyl (DPPH) and ferric reducing antioxidant power (FRAP) were evaluated in both the skins and pulps of the grapes, along with the polyphenolic profile in the skins.

### 2.4. Total Phenolic Compounds

Determination was carried out using the Folin–Ciocalteu method [12]. Approximately 2 g of grape skin or pulp was extracted with 20 mL of 50% acetone for 30 min at room temperature, followed by ultrasonic bath treatment for 15 min. The extracts were then centrifuged at 4000 rpm for 10 min at 4 °C. Quantification was performed using a spectrophotometer at 725 nm, employing a gallic acid standard curve and expressing results as mg of gallic acid equivalent per 100 g of skin or pulp. The reaction consisted of 100 µL of extract, 500 µL of Folin–Ciocalteu reagent (previously diluted 1:10 with distilled water), 1.5 mL of 7.5% sodium carbonate solution, and 8.4 mL of distilled water. The mixture was incubated at room temperature for 30 min before reading the absorbance.

### 2.5. Total Flavonoids

Determination followed the method of [13], with adaptations [14]. Approximately 2 g of grape skin or pulp was extracted with 20 mL of acidified methanol (methanol with 1% HCl) for 30 min at room temperature, followed by centrifugation at 4000 rpm for 10 min at 4 °C. Absorbance was measured at 425 nm, and results were expressed as mg equivalent of quercetin per 100 g of skin or pulp. The reaction mixture contained 500 µL of extract, 1.5 mL of 5% aluminum chloride solution, and 8.5 mL of distilled water. The samples were incubated at room temperature for 30 min before absorbance measurement.

### 2.6. Total Monomeric Anthocyanins

Determination was conducted using the differential pH method [15]. Approximately 2 g of grape skin or pulp was extracted with 20 mL of acidified methanol (methanol with 1% HCl) for 30 min at room temperature, followed by centrifugation at 4000 rpm for 10 min at 4 °C. Samples were then homogenized in buffers of pH 1.0 and 4.5. Absorbance was measured at 510 and 700 nm, with results expressed as mg equivalent of cyanidin-3-glucoside per 100 g of skin or pulp. The final absorbance (A) was calculated using the formula: A = (A510 − A700)pH 1.0 − (A510 − A700)pH 4.5, and the concentration was determined using a molar extinction coefficient of 26,900 L·mol^−1^·cm^−1^ for cyanidin-3-glucoside.

### 2.7. Antioxidant Activity

Antioxidant activity was assessed using the DPPH and FRAP methods. For the DPPH assay [16], approximately 2 g of grape skin or pulp was extracted with 20 mL of 50% methanol for 30 min at room temperature, followed by centrifugation at 4000 rpm for 10 min at 4 °C. The reaction mixture contained 100 µL of extract and 3.9 mL of 0.1 mM DPPH solution in methanol. The samples were incubated in the dark for 30 min at room temperature, and absorbance was measured at 517 nm. Results were expressed as mmol equivalent TEAC per g of skin or pulp.

For the FRAP assay [17], approximately 2 g of grape skin or pulp was extracted with 20 mL of 50% methanol for 30 min at room temperature, followed by centrifugation at 4000 rpm for 10 min at 4 °C. The reaction mixture contained 100 µL of extract, 3 mL of freshly prepared FRAP reagent (300 mM acetate buffer, 10 mM TPTZ in 40 mM HCl, and 20 mM FeCl3 in a 10:1:1 ratio), and 100 µL of distilled water. The samples were incubated at 37 °C for 30 min, and absorbance was measured at 593 nm. Results were expressed as mmol Fe per kg of skin or pulp.

### 2.8. Phenolic Compounds in Grape Skins by Ultra-High-Performance Liquid Chromatography (UHPLC)

Phenolic compounds were identified and quantified via high-performance liquid chromatography (HPLC) (Thermo Fisher Scientific, Bremen, Germany) [18], using an extraction solvent of acetone:methanol:water:acetic acid (42:30:27.5:0.5, *v*/*v*). Approximately 2 g of grape skin was extracted with 20 mL of solvent for 30 min at room temperature, followed by centrifugation at 4000 rpm for 10 min at 4 °C. Samples were filtered (0.45 μm), injected into a UHPLC system (Ultimate 3000 BioRS, Dionex-Thermo Fisher Scientific, Sunnyvale, CA, USA), equipped with a DAD detector and a Luna^®^ C18 column, using a mobile phase gradient of 0.85% phosphoric acid (A) and acetonitrile (B). The gradient program was as follows: 0–5 min, 5% B; 5–15 min, 5–25% B; 15–30 min, 25–50% B; 30–35 min, 50–5% B; 35–40 min, 5% B. The flow rate was 1.0 mL/min, the injection volume was 20 µL, and the detection was performed at 280 nm for phenolic acids, 320 nm for flavonols, and 520 nm for anthocyanins. Identification was performed by comparison with commercial standards (≥95% purity, Sigma-Aldrich, St. Louis, MO, USA), and quantification was achieved using calibration curves, expressed in mg per kg of skin.

### 2.9. Statistical Analyses

The data were subjected to analysis of variance (ANOVA) to evaluate the effects of rootstocks and training systems, as well as their interactions. Subsequently, mean comparisons were performed using Tukey’s test (*p* < 0.05), employing the Sisvar 6.0 software.

For principal component analysis (PCA), singular value decomposition (SVD) was applied, considering the joint analysis of the *Vitis labrusca* varieties ‘Bordô’ and ‘Isabel’, as well as the hybrids ‘IAC 138-22 Máximo’ and ‘BRS Violeta’. The PCA was conducted using Unscrambler^®^ X software (Version 10.4).

## 3. Results

### 3.1. Grape Vines Vitis labrusca—‘Bordô’

Regarding the results obtained for both skin and pulp, it is observed that, predominantly, with the exception of total flavonoids (TF) for skin, all concentrations were higher in the High Trellis system compared to the Low Trellis system for the rootstock ‘IAC 766 Campinas’. The same pattern was observed for ‘106-8 Mgt’, but only for skin. For the pulp, higher levels were obtained in the Low Trellis system for TF and DPPH (Table 1).

**Figure 1:**
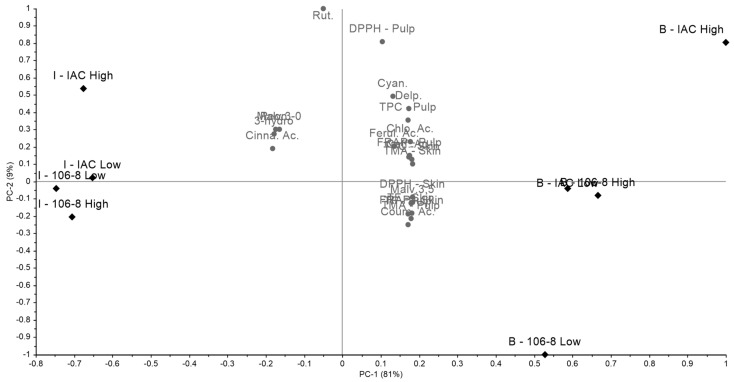
Principal component analysis (PCA) of the biochemical parameters and phenolic compound profile of ‘Bordô’ and ‘Isabel’ grapes under different training systems and rootstocks. I-IAC Low—‘Isabel’, ‘IAC 766 Campinas’, low trellis; I-IAC High—‘Isabel’, ‘IAC 766 Campinas’, high trellis; I-106-8 Low—‘Isabel’, ‘106-8 Mgt’, low trellis; I-106-8 High—‘Isabel’, ‘106-8 Mgt’, high trellis; B-IAC Low—‘Bordô’, ‘IAC 766 Campinas’, low trellis; B-IAC High—‘Bordô’, ‘IAC 766 Campinas’, high trellis; B-106-8 Low—‘Bordô’, ‘106-8 Mgt’, low trellis; B-106-8 High—‘Bordô’, ‘106-8 Mgt’, high trellis. TF: total flavonoids; TPC: total phenolic compounds; TMA: total monomeric anthocyanins; Malv.3,5: malvidin-3,5-diglycoside; Delp.: delphinidin-3-O-glucoside; Cyan.: Cyanidin-3-O-glucoside; Peon.: peonidin-3-O-glucoside; Malv.3-0: malvidin-3-O-glucoside; Rut.: rutin; 3-hydro – 3-hydroxytyrosol acid; Caff. Ac.: caffeic acid; Chlo. Ac.: chlorogenic acid; Coum. Ac.: *p*-coumaric acid; Cinna. Ac.: *t*-cinnamic acid; Ferul. Ac.: *t*-ferulic acid.

When considering the two rootstocks for the skin analysis, a statistical difference was found only for the variables TF, total monomeric anthocyanins (TMA), and total phenolic compounds (TPC) in the High Trellis system. TF was higher with the use of rootstock ‘106-8 Mgt’ (95.88 mg 100 g^−1)^ compared to ‘IAC 766 Campinas’ (81.23 mg 100 g^−1^); however, the opposite was observed for TMA and TPC, with ‘IAC 766 Campinas’ being superior (835.01 and 956.12 mg 100 g^−1^, respectively) compared to ‘106-8 Mgt’ (680.04 and 804.17 mg 100 g^−1^, respectively) (Table 1). For the pulp, the rootstock ‘IAC 766 Campinas’ stood out, showing higher levels than those obtained in ‘106-8 Mgt’ for TF (High Trellis), TMA (High Trellis), and DPPH (both High and Low Trellis). Conversely, the levels of TF and TMA in the Low Trellis system for ‘106-8 Mgt’ were higher than those obtained in ‘IAC 766 Campinas’ (Figure 1). It is noteworthy that the concentrations of these variables found for pulp are drastically lower than those obtained for skin, as expected.

In the variables that did not show interaction with each other and were analyzed individually (Table 2), it was found that for the FRAP of the skin, there was no statistical difference. However, a statistical difference was found between the rootstocks for TPC and FRAP of the pulp, with ‘IAC 766 Campinas’ presenting the highest levels (42.76 mg 100 g^−1^ and 16.15 mmol Fe kg^−1^) (Table 2).

The quantification of individual phenolic compounds by UHPLC revealed a significant interaction between the training systems and rootstocks. The combination of the high trellis with the ‘IAC 766 Campinas’ rootstock yielded the highest concentrations of the anthocyanins delphinidin-3-O-glucoside (20.24 mg kg^−1^), cyanidin-3-O-glucoside (2.21 mg kg^−1^), and peonidin-3-O-glucosidelucoside (1.37 mg kg^−1^) (Table 3), corroborating the greater accumulation of TMA (835.01 mg 100 g-1 of skin) (Table 1). Conversely, the combination of the high trellis with the ‘106-8 Mgt’ rootstock showed higher concentrations of malvidin-3.5-diglucoside (157.33 mg kg^−1^) and malvidin-3-O-glucoside (7.90 mg kg^−1^).

Considering both rootstocks and trellising, it was noted that, with the exception of the compound malvidin-3-O-glucoside, all others showed significantly higher levels when grown on a high trellis. Furthermore, the values obtained for ‘IAC 766 Campinas’ were greater compared to those for the ‘106-8Mgt’, with superior concentrations for delphinidin-3-O-glucoside on both low (18.02 mg kg^−1^) and high trellises (20.24 mg kg^−1^); cyanidin-3-O-glucoside on a high trellis (2.21 mg kg^−1^); peonidin-3-O-glucoside on a low (1.28 mg kg^−1^) and high trellis (1.37 mg kg^−1^); and malvidin-3-O-glucoside on a low trellis (7.74 mg kg^−1^). Only for malvidin-3,5-diglycoside on a high trellis (157.33 mg kg^−1^) and malvidin-3-O-glucoside on a high trellis (7.90 mg kg^−1^) were higher concentrations observed for the 106-8 ‘Mgt’ rootstock.

The rutin (flavonol) content in the skins ranged from 6.76 to 9.64 mg kg^−1^, with the high trellis promoting the highest concentrations, regardless of the rootstock (Table 3).

Regarding the profile of phenolic acids, the two major compounds were caffeic acid, varying from 2.50 to 4.51 mg kg^−1^, and chlorogenic acid, with concentrations ranging from 3.13 to 4.17 mg kg^−1^. The combination of the high trellis with both rootstocks showed higher contents, particularly for the ‘106-8 Mgt’ for caffeic acid and the ‘IAC 766 Campinas’ for chlorogenic acid (Table 3).

The other two identified compounds, *p*-coumaric acid and *t*-ferulic acid, had lower concentrations. For *p*-coumaric acid, there was no statistical difference between the high and low trellis with the ‘IAC 766 Campinas’ rootstock, but a significant difference was observed with the ‘106-8 Mgt’, where the high trellis showed a higher content (0.45 mg kg^−1^). For *t*-ferulic acid, a higher content was obtained for the high trellis with ‘IAC 766 Campinas’ (1.20 mg kg^−1^), in contrast to the ‘106-8 Mgt’, where a higher content was found in the low trellis (1.18 mg kg^−1^). In the total analysis of the detected compounds (Table 3), higher levels were obtained for the high trellis in both rootstocks, with no statistical difference between them (‘IAC 766 Campinas’—201.11 mg kg^−1^ and ‘106-8 Mgt’—206.48 mg kg^−1^).

### 3.2. Grape Vines Vitis labrusca—‘Isabel’

The training systems and rootstocks independently influenced the bioactive compounds and antioxidant activity of ‘Isabel’ grapes (Table 4). The high trellis led to higher TPC and greater antioxidant activity (DPPH and FRAP) in the skins. Among the rootstocks, ‘IAC 766 Campinas’ exhibited higher TPC and TF in the skins compared to ‘106-8 Mgt’. In the pulp, the high trellis increased TF content, while ‘106-8 Mgt’ had higher TF than ‘IAC 766 Campinas’. No significant differences were observed in DPPH antioxidant activity in the pulp.

There was an interaction between the rootstocks and training systems for the TMA in both the skins and pulp, as well as for the TPC, TMA, and FRAP in the pulp (Table 5). It was found that the TMA was higher for the high trellis, especially with the rootstock ‘IAC 766 Campinas’ (166.02 mg 100 g^−1^) for the skins. In the case of the pulp, the high trellis showed superior values for all variables (TPC, TMA, and FRAP) for ‘IAC 766 Campinas’. However, no statistical differences were found for these variables with the ‘106-8 Mgt’. Furthermore, regarding the rootstocks, it is noted that, with the exception of TPC for the low trellis, all means were higher, showing significant differences for the rootstock ‘IAC 766 Campinas’ compared to the ‘106-8 Mgt’ (Table 5).

In the analysis of phenolic compounds using UHPLC, an interaction between training systems and rootstocks was also recorded (Table 6). Regarding the concentration of anthocyanins specifically, the following order was observed for the skins of ‘Isabel’ grapes: malvidin-3-O-glucoside > delphinidin-3-O-glucoside > malvidin-3,5-diglycoside > peonidin-3-O-glucoside > cyanidin-3-O-glucoside. It was found that, with the exception of the compound cyanidin-3-O-glucoside for the rootstock ‘106-8 Mgt’, all others were found to have significantly higher levels when grown on a high trellis. Regarding the rootstocks, all compounds were found to have higher levels in ‘IAC 766 Campinas’ compared to ‘106-8 Mgt’.

In the analysis of the only detected flavonol, rutin, divergent levels were obtained between the trellises and rootstocks. For ‘IAC 766 Campinas’, the High Trellis system (10.24 mg kg^−1^) showed a higher level than the Low Trellis system (8.58 mg kg^−1^), while for ‘106-8 Mgt’, the opposite was observed, with a higher level in the Low Trellis system (9.89 mg kg^−1^) compared to the High Trellis system (9.00 mg kg^−1^).

Among the phenolic acids found, there was substantial variation between high and low trellises. For the rootstocks ‘IAC 766 Campinas’ and ‘106-8 Mgt’, the high trellis was superior for the compounds 3-hydroxytyrosol acid, *t*-cinnamic acid, and *t*-ferulic acid, as well as chlorogenic acid, only for ‘IAC 766 Campinas’. For the other compounds, higher content was observed in the high trellis (Table 6). Regarding the rootstocks, ‘IAC 766 Campinas’ stood out with higher concentrations for chlorogenic acid (both low and high trellises), *p*-coumaric acid, *t*-cinnamic acid, and *t*-ferulic acid (low trellis) (Table 6).

Overall, analyzing all compounds, higher levels were observed for both rootstocks on the high trellis, with a statistical difference between ‘IAC 766 Campinas’ (77.31 mg kg^−1^) and ‘106-8 Mgt’ (66.01 mg kg^−1^) (Table 6).

### 3.3. Principal Component Analysis (PCA) of Vitis labrusca Grapevines

In the principal component analysis (PCA) for all variables, considering the two varieties ‘Bordô’ and ‘Isabel’, along with their respective rootstocks ‘IAC 766 Campinas’ and ‘106-8 Mgt’, in both low and high trellis systems, for both skin and pulp, a total variance of 90% was observed, with PC1 representing 81% (Figure 1). A clear distinction was noted between the varieties ‘Bordô’ (B-IAC Low, B-IAC High, B-106-8 Low, and B-106-8 High) and ‘Isabel’ (I-IAC Low, I-IAC High, I-106-8 Low, and I-106-8 High), with ‘Bordô’ and ‘Isabel’ separated in the right and left quadrants, respectively.

It was observed that predominantly (82%), the variables, including both biochemical parameters and phenolic profiles, were grouped with the cultivar ‘Bordô’. Thus, for both skin and pulp, total phenolics (TF), total phenolic compounds (TPC), total monomeric anthocyanins (TMA), antioxidant activities (DPPH and FRAP), as well as malvidin-3,5-diglucoside, delphinidin-3-O-glucoside, cyanidin-3-O-glucoside, rutin, caffeic acid, chlorogenic acid, *p*-coumaric acid, and *t*-ferulic acid, were grouped in the right quadrant (PC1 +, PC1 -). Only the variables peonidin-3-O-glucoside, malvidin-3-O-glucoside, 3-hydroxytyrosol acid, and *t*-cinnamic acid were grouped with the variety ‘Isabel’. It is worth noting that no distinctions were observed between high and low trellis systems in general, nor was there any separation between the rootstocks ‘IAC 766 Campinas’ and ‘106-8 Mgt’.

### 3.4. Hybrid Vines—‘IAC 138-22 Máximo’

An interaction was observed between the rootstocks and training systems for the levels of TF, TPC, TMA, and antioxidant activity (DPPH) in the skin, as well as for TMA in the pulp of the ‘IAC 138-22 Máximo’ grape (Table 7). For TF, there was only a statistical difference for the rootstock ‘106-8 Mgt’ in high trellis (136.23 mg 100 g^−1^) compared to low (95.07 mg 100 g^−1^), which was also the case for TMA, where this sample was higher (605.89 mg 100 g^−1^). Regarding TPC, the high trellis showed higher levels for both rootstocks, differing statistically, with the maximum obtained being 1059.67 mg 100 g^−1^ in the rootstock ‘IAC 766 Campinas’. Similarly, for DPPH in the skin, higher levels were obtained in the high trellis for both ‘IAC 766 Campinas’ (24.49 mmol g^−1^) and ‘106-8 Mgt’ (25.58 mmol g^−1^).

For the pulp, in TMA, a statistical difference was observed between high (2.06 mg 100 g^−1^) and low (1.29 mg 100 g^−1^) trellis for ‘IAC 766 Campinas’, but not for ‘106-8 Mgt’. However, among the rootstocks, higher levels were verified in both low (2.66 mg 100 g^−1^) and high (2.64 mg 100 g^−1^) trellis for ‘106-8 Mgt’ (Table 7). Thus, it is again evident that the high trellis is capable of promoting a greater increase in TF, TPC, TMA, and antioxidant compounds.

Regarding FRAP for the skin and TF, TPC, DPPH, and FRAP for the pulp, there was no significant interaction between the factors. Thus, they were analyzed separately (Table 8). In the case of FRAP for the skin, a statistical difference was obtained only when considering the rootstock, where ‘106-8 Mgt’ stood out (304.26 mmol Fe kg^−1^). For the pulp, significant differences were observed in opposite directions when considering TF and FRAP in relation to TPC and DPPH. For TF (1.31 mg 100 g^−1^) and FRAP (9.70 mmol Fe kg^−1^), the levels were higher in the high trellis; however, TPC (16.96 mg 100 g^−1^) and DPPH (0.30 mmol g^−1^) were higher in the low trellis. Regarding the rootstocks, ‘IAC 766 Campinas’ showed higher levels of TF (1.36 mg 100 g^−1^), DPPH (0.39 mmol g^−1^), and FRAP (12.89 mmol Fe kg^−1^). There was no statistical difference for TPC (Table 8).

There was an interaction between the training systems and the rootstocks for all the individual phenolic compounds quantified by UHPLC. Among the quantified anthocyanins, malvidins showed the highest concentrations, with malvidin-3,5-diglucoside being the predominant one, varying between 106.10 and 152.45 mg kg^−1^ in the skin, followed by malvidin-3-O-glucoside, which ranged from 28.79 to 43.91 mg kg^−1^. The combination of low trellis and rootstock ‘106-8 Mgt’ resulted in the highest concentrations of all identified anthocyanins, including when compared to the skins of the ‘IAC 138-22 Máximo’ grape (Table 9).

Rutin was found at a higher concentration in the combination of low trellis and rootstock ‘106-8 Mgt’ (29.07 mg kg^−1^) (Table 9). Among the phenolic acids, chlorogenic acid was the major compound, presenting higher concentrations in the combination of high trellis and rootstock ‘IAC 766 Campinas’ (3.43 mg kg^−1^), which also resulted in a greater accumulation of caffeic acid (1.46 mg kg^−1^). Overall, the high trellis was superior to the low trellis for all identified compounds in rootstock ‘IAC 766 Campinas’. However, the opposite was observed for ‘106-8 Mgt’, where, with the exception of 3-hydroxytyrosol acid, all compounds were higher in the low trellis. Thus, the results were significantly different among the rootstocks, with higher content for high trellis for ‘IAC 766 Campinas’ and higher content for low trellis for ‘106-8 Mgt’ (Table 9).

Finally, the same pattern observed for total phenolics was confirmed for catechin, where the high trellis associated with rootstock ‘IAC 766 Campinas’ also resulted in the highest levels (2.76 mg kg^−1^), contrary to what was found for the association between the Low Trellis system and rootstock ‘106-8 Mgt’ (2.42 mg kg^−1^) (Table 9). Considering all the compounds found, a higher content was obtained with the low trellis and rootstock ‘106-8 Mgt’ (272.29 mg kg^−1^) (Table 9).

### 3.5. Hybrid Vines—‘BRS Violeta’

In the analysis of the hybrid variety ‘BRS Violeta’, it was found that there was no interaction for the variables TF and DPPH for skin and DPPH for pulp (Table 10). For the TF of the skin, a statistical difference was obtained considering the trellis, with higher levels in the high trellis (167.97 mg 100 g^−1^), as well as between the rootstocks, with ‘106-8 Mgt’ being superior (156.42 mg 100 g^−1^). In the DPPH of the skin, a statistical difference was found only between the rootstocks, with ‘IAC 766 Campinas’ (130.57 mmol g^−1^) standing out. Conversely, no statistical difference was found in the DPPH of the pulp between the rootstocks, but the high trellis (0.72 mmol g^−1^) was significantly superior to the low trellis (0.60 mmol g^−1^).

Regarding the levels of TPC, TMA, and FRAP for the skin, as well as TF, TPC, TMA, and FRAP for the pulp, a correlation was found between the rootstocks and the trellis (Table 11). For the skin, higher levels of TPC, TMA, and FRAP were obtained in the high trellis for ‘IAC 766 Campinas’ and TPC for ‘106-8 Mgt’. Among the rootstocks, ‘106-8 Mgt’, except for TMA (high trellis), which showed no statistical difference, stood out for all other variables, with higher levels that differed statistically.

For the pulp, the results were divergent, with TF, TMA, and FRAP being higher in the high trellis and TPC in the low trellis for ‘IAC 766 Campinas’. On the other hand, TF and TMA were higher in the low trellis, while TPC and FRAP were higher in the high trellis for ‘106-8 Mgt’. Among the rootstocks, ‘IAC 766 Campinas’ stood out, showing higher content for TF (high trellis), TPC (low trellis), and TMA (both low and high trellis). Only FRAP (low trellis) was higher for the rootstock ‘106-8 Mgt’ (Table 11).

Regarding the profile of phenolic compounds obtained by UHPLC, it was observed that among the anthocyanins, malvidin-3,5-diglycoside stood out for the rootstock ‘IAC 766 Campinas’, with a variation of 539.06 to 665.00 mg kg^−1^. For ‘106-8 Mgt’, higher levels were obtained for delphinidin-3-O-glucoside, which varied from 177.01 to 198.85 mg kg^−1^, as well as for cyanidin-3-O-glucoside, with a variation from 164.24 to 199.44 mg kg^−1^. However, it is noted that the maximum obtained for an anthocyanin in ‘106-8 Mgt’ (199.44 mg kg^−1^) is 70% lower than the maximum obtained for ‘IAC 766 Campinas’ (665.00 mg kg^−1^). All anthocyanins showed higher values when in the high trellis, differing statistically from the low trellis. Among the rootstocks, except for malvidin-3-O-glucoside (both low and high trellis), all levels were higher in ‘IAC 766 Campinas’ (Table 12).

The levels of rutin were contrary regarding both the trellis and the rootstock, with higher levels in the low trellis for ‘IAC 766 Campinas’ (10.91 mg kg^−1^), while in ‘106-8 Mgt’, a higher level was obtained in the high trellis (15.31 mg kg^−1^) (Table 12).

### 3.6. Principal Component Analysis (PCA) of Hybrid Vines

**Figure 2:**
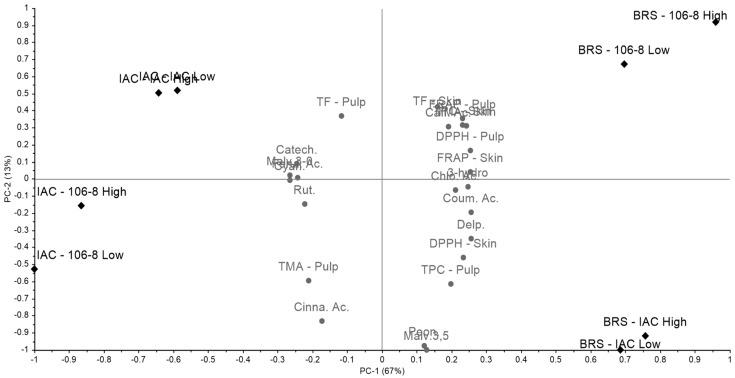
Principal component analysis (PCA) of the biochemical parameters and phenolic compound profile of ‘IAC 138-22 Máximo’ and ‘BRS Violeta’ grapes under different training systems and rootstocks. IAC-106-8 Low–‘IAC 138-22 Máximo’, ‘IAC 766 Campinas’, low trellis; IAC-106-8 High – ‘IAC 138-22 Máximo’, ‘IAC 766 Campinas’, high trellis; IAC-106-8 Low–IAC 138-22 Máximo’, ‘106-8 Mgt’, low trellis; IAC-106-8 High–IAC 138-22 Máximo’, ‘106-8 Mgt’, high trellis; BRS-IAC Low—‘BRS Violeta’, ‘IAC 766 Campinas’, low trellis; BRS-IAC High—‘BRS Violeta’, ‘IAC 766 Campinas’, high trellis; BRS-106-8 Low—‘BRS Violeta’, ‘106-8 Mgt’, low trellis; BRS-106-8 High—‘BRS Violeta’, ‘106-8 Mgt’, high trellis. TF: total flavonoids; TPC: total phenolic compounds; TMA: total monomeric anthocyanins; Malv.3,5: malvidin-3,5-diglycoside; Delp.: delphinidin-3-O-glucoside; Cyan.: Cyanidin-3-O-glucoside; Peon.: peonidin-3-O-glucoside; Malv.3-0: malvidin-3-O-glucoside; Rut.: rutin; 3-hydro: 3-hydroxytyrosol acid; Caff. Ac.: caffeic acid; Chlo. Ac.: chlorogenic acid; Coum. Ac.: *p*-coumaric acid; Cinna. Ac.: *t*-cinnamic acid; Ferul. Ac.: *t*-ferulic acid. Catech.: catechin.

In the PCA considering all variables for the hybrid varieties ‘IAC 138-22 Máximo’ and ‘BRS Violeta’, along with their respective rootstocks ‘IAC 766 Campinas’ and ‘106-8 Mgt’, in both low and high trellis systems, for both skin and pulp, a total variance of 80% was obtained, with PC1 representing 67% (Figure 2). Similarly, to what was observed in the PCA of the varieties ‘Bordô’ and ‘Isabel’, a separation between the two hybrid varieties was noted, with ‘IAC 138-22 Máximo’ on the left in the quadrants and ‘BRS Violeta’ on the right. In this case, 70% of the variables were grouped in the quadrants of ‘BRS Violeta’ (BRS-IAC Low, BRS-IAC High, BRS-106-8 Low, BRS-106-8 High), while 30% were grouped with ‘IAC 138-22 Máximo’ (IAC-IAC Low, IAC-IAC High, IAC-106-8 Low, IAC-106-8 High).

**Figure 3:**
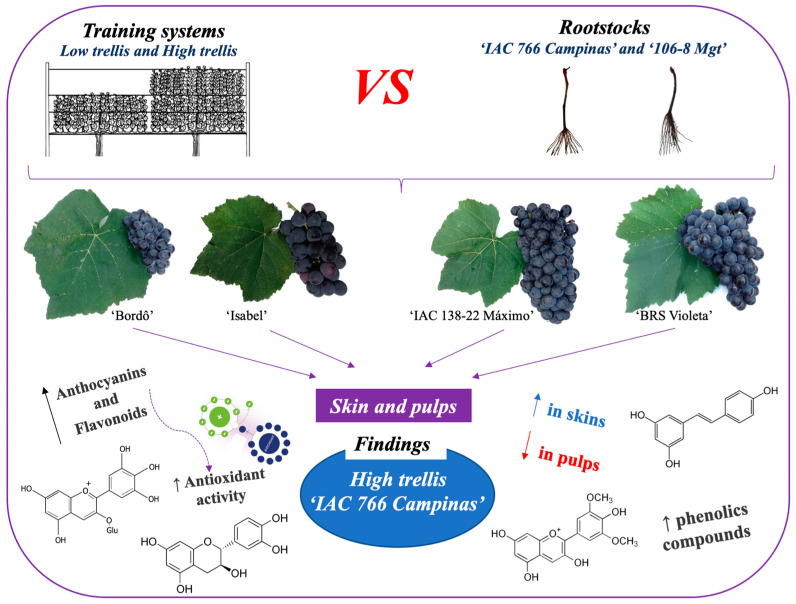
Graphical abstract of training systems (low and high trellis) and rootstocks (‘IAC 766 Campinas’ and ‘106-8 Mgt’) affect the phenolic composition and antioxidant activity in skins and pulps of hybrid grapes (‘IAC 138-22 Máximo’ and ‘BRS Violeta’) and *Vitis labrusca* (‘Bordô’ and ‘Isabel’).

The PCA showed a separation between the rootstocks and trellis systems (low and high) for each variety. ‘BRS Violeta’ with rootstock ‘106-8 Mgt’, in both the low and high trellises, was found in PC1+, while ‘BRS Violeta’ with rootstock ‘IAC 766 Campinas’, in both the low and high trellises, was found in PC1−. Similarly, a comparable profile was observed for ‘IAC 138-22 Máximo’, with rootstock ‘IAC 766 Campinas’, in both the low and high trellises, appearing in PC2+, in contrast to rootstock ‘106-8 Mgt’, in both the low and high trellises, found in PC2− (Figure 2 and Figure 3).

## 4. Discussion

The interaction between the training system and rootstock significantly impacts the biochemical composition of grapes. Therefore, selecting the appropriate training system and rootstock that enhance compatibility with the grafted cultivar is crucial for maximizing the accumulation of antioxidant compounds in the grapes.

Overall, it is evident that the high trellis shows greater efficiency in promoting the accumulation of important compounds in grapes. This effect is likely linked to an increased photosynthetic rate in the vines [11], which enhances energy and sugar accumulation, ultimately favoring the synthesis of antioxidant compounds [4,7,11,19]. This response can be attributed to the larger leaf area provided by high trellis systems, as reported in other studies, which increases light interception and promotes a higher carbon assimilation rate, supporting the synthesis of secondary metabolites [4,11]. Additionally, the greater canopy volume likely improves microclimatic conditions, reducing temperature extremes and enhancing photosynthetic efficiency [11].

This process contributes to increased energy accumulation and, consequently, higher concentrations of bioactive compounds. Based on the results obtained, the combination of the high trellis with the ‘IAC 766 Campinas’ rootstock led to the highest accumulation of compounds, making it recommended for the cultivation of ‘Bordô’ grapevines, as it provides higher concentrations of compounds with antioxidant potential in the grapes, which consequently will affect the beverages, as observed by Domingues Neto et al. (2024) [19]. This can be attributed to the larger leaf area typically developed in high trellis systems, which increases light interception and photosynthetic activity, enhancing carbohydrate production and subsequent synthesis of secondary metabolites, as previously demonstrated for this rootstock and training system combination [5,11].

Furthermore, the results indicate a strong influence of rootstocks on the anthocyanin biosynthetic pathway in ‘BRS Violeta’ grapes, a phenomenon also observed in previous studies with this variety grafted onto the ‘IAC 766 Campinas’ and ‘106-8 Mgt’ rootstocks [20]. Although few studies have evaluated the effect of rootstocks on hybrid grapes intended for juice and wine production, research on other *Vitis vinifera* varieties, such as ‘Red Alexandria’ [6] and ‘Greco Nero’ [21], has also reported rootstock effects on anthocyanin content. In particular, rootstocks have been shown to influence the availability of water and nutrients to the scion, affecting both vegetative growth and the synthesis of phenolic compounds in the berries [4,5,6,7,8,9,11].

Although the ‘Isabel’ grape is a *Vitis labrusca*, a species in which diglycosylated anthocyanins are predominant [22], the main anthocyanin found in the ‘Isabel’ grapes of the present study was malvidin-3-O-glucoside, corroborating previous studies [23,24]. This result may be related to the genetic background of ‘Isabel’, which, despite being a *V. labrusca* cultivar, shares phenotypic traits with hybrid varieties that can accumulate significant amounts of malvidin-3-O-glucoside, particularly under specific environmental and agronomic conditions [20,22,23]. Furthermore, it was generally observed that the combination of high trellising and the ‘IAC 766 Campinas’ rootstock demonstrated an ability to elevate the electron transport rate (ETR) of the vines [11], with higher values indicating better photosynthetic activity, which consequently influenced the quality of the current genotype. Therefore, in addition to the vines being more photosynthetically active, they developed with lower stress levels, improving fruit quality through greater synthesis of carbohydrates and ATP, as well as reduced energy expenditure in the synthesis and activation of the antioxidant system, resulting in increased concentrations of total and individual anthocyanins identified in the berries and juices of the ‘Isabel’ grape [19].

Notably, higher levels of phenolic compounds, flavonoids, and antioxidant activity are found in grape skins compared to the pulp. This difference can be attributed to the role of the skin as a protective barrier, which necessitates the accumulation of secondary metabolites to defend against environmental stressors such as ultraviolet radiation, pathogens, and oxidative damage [25,26,27]. The synthesis of anthocyanins and flavonoids in the skin is particularly stimulated by light exposure, leading to greater concentrations of these bioactive compounds than in the pulp [28]. Additionally, the pulp primarily serves as a carbohydrate storage tissue, containing lower levels of these antioxidant compounds [29]. This difference in the distribution of bioactive compounds between skin and pulp is a critical factor influencing the overall antioxidant capacity of grape-derived products, as the skin contributes significantly to the phenolic content of both wines and juices [9,18,19,20].

The accumulation of phenolic compounds in berries is influenced by environmental and stress factors, particularly exposure to ultraviolet radiation, which can activate genes responsible for their synthesis in secondary metabolism [30]. This phenomenon explains the higher levels of phenolic compounds observed in low trellis systems, which enhance light exposure for the clusters and, consequently, promote greater accumulation of these bioactive substances. However, the increased light exposure in low trellis systems can also lead to higher oxidative stress in the berries, potentially reducing the overall antioxidant capacity if not balanced by adequate water and nutrient availability [5,11]. These phenolic compounds exhibit significant biological activity, offering protection against various diseases associated with oxidative stress [31,32]. Additionally, they contribute to preventing aging and collagen degradation [33] and play a role in managing type 2 diabetes mellitus [34,35]. Thus, grapes are recognized as an important functional food in addressing various health issues. Consequently, if the choice of cultivation system prioritizes these benefits, the low trellis system should be given serious consideration.

Regarding phenolic acids, it is worth noting that chlorogenic acid was the predominant phenolic acid in the skins, corroborating studies investigating juices from ‘Isabel’ grapes [19]. This compound is associated with antioxidant properties and tissue repair processes [36]. Its high concentration in skins compared to pulps further supports the critical role of berry skins in determining the antioxidant potential of grape-derived products.

In general, the principal component analysis (PCA) supported these findings, confirming that ‘BRS Violeta’ grapes have a higher content of phenolic compounds and greater antioxidant activity, consistent with previous studies [20,37,38]. Among the tested combinations, the ‘IAC 766 Campinas’ rootstock proved to be the most effective, likely due to its superior vigor compared to the ‘106-8 Mgt’ [39,40]. This superior vigor likely enhances the accumulation of secondary metabolites by promoting greater biomass production and nutrient uptake, factors known to positively influence phenolic synthesis in grape berries [4,5,9,11,19,20].

The combination of the hybrid varieties ‘IAC 138-22 Máximo’ and ‘BRS Violeta’ represents a promising alternative for grape growers and processing industries. While ‘IAC 138-22 Máximo’ is notable for its high productivity [5], ‘BRS Violeta’ offers a higher content of bioactive compounds. The synergy between these varieties can help balance their limitations and meet the standards required by Brazilian legislation for juice and wine production, resulting in beverages with high antioxidant potential. These results reinforce the importance of selecting the appropriate rootstock and training system combination to optimize grape quality for industrial applications.

## 5. Conclusions

Rootstocks and training systems significantly influence the accumulation of phenolic compounds and antioxidant activity in grapes. For hybrid cultivars, a high trellis combined with the ‘106-8 Mgt’ rootstock enhances antioxidant compound accumulation, while in *Vitis labrusca*, its interaction with ‘IAC 766 Campinas’ increases flavonoid, anthocyanin, and phenolic acid concentrations. Among *Vitis labrusca* varieties, ‘Bordô’ exhibits the highest levels of bioactive compounds, whereas ‘Isabel’ is notable for its specific phenolic acids. Therefore, selecting an appropriate training system and rootstock combination is crucial for optimizing grape quality and bioactive compound content.

## Figures and Tables

**Table 1:** Total flavonoids (TF), total monomeric anthocyanins (TMA), and antioxidant activity (DPPH) of the skin and pulp, along with total phenolic compounds (TPC), in the skin of the ‘Bordô’ grape under different training systems and rootstocks.

Variable	Trellis	Rootstock		CV (%)
		‘IAC 766 Campinas’	‘106-8 Mgt’	
Skin				
TF (mg 100 g^−1^)	Low	85.98 ± 9.93 aA	78.58 ± 4.45 bA	7.15
	High	81.23 ± 3.12 aB	95.88 ± 4.60 aA	
TMA (mg 100 g^−1^)	Low	513.66 ± 21.91 bA	569.96 ± 52.99 bA	6.33
	High	835.01 ± 38.32 aA	680.04 ± 44.79 aB	
DPPH (mmol g^−1^)	Low	17.33 ± 3.07 bA	20.03 ± 0.84 aA	9.04
	High	22.67 ± 1.35 aA	20.15 ± 1.08 aA	
TPC (mg 100 g^−1^)	Low	679.80 ± 41.41 bA	719.59 ± 39.64 aA	7.00
	High	956.12 ± 49.11 aA	804.17 ± 56.06 aB	
Pulp				
TF (mg 100 g^−1^)	Low	0.76 ± 0.07 bB	0.89 ± 0.03 aA	5.78
	High	0.92 ± 0.01 aA	0.72 ± 0.05 bB	
TMA (mg 100 g^−1^)	Low	4.92 ± 0.24 aB	5.59 ± 0.34 aA	5.51
	High	5.32 ± 0.25 aA	3.84 ± 0.22 bB	
DPPH (mmol g^−1^)	Low	1.12 ± 0.03 bA	0.35 ± 0.02 aB	21.85
	High	2.02 ± 0.42 aA	0.36 ± 0.02 aB	

Means followed by the same lowercase letter in the column and uppercase letter in the row do not differ from each other according to the Tukey test at a 5% probability level. CV: Coefficient of Variation.

**Table 2:** Antioxidant activity (FRAP) of the skin and pulp and total phenolic compounds (TPC) of the pulp of ‘Bordô’ grapes in different training systems and rootstocks.

Variable	Trellis		Rootstock		CV (%)
	Low	High	‘IAC 766 Campinas’	‘106-8 Mgt’	
Skin					
FRAP (mmol Fe kg^−1^)	257.10 ± 27.23 a	285.20 ± 37.12 a	258.31 ± 41.21 a	284.00 ± 22.25 a	11.63
Pulp					
TPC (mg 100 g^−1^)	33.40 ± 10.66 a	40.57 ± 9.05 a	42.76 ± 6.55 a	31.21 ± 10.29 b	20.97
FRAP (mmol Fe kg^−1^)	14.42 ± 1.59 a	14.60 ± 3.22 a	16.15 ± 1.74 a	12.87 ± 1.93 b	9.64

Means followed by the same lowercase letter in the row, within the same factor, do not differ from each other according to the Tukey test at a 5% probability level. CV: Coefficient of Variation.

**Table 3:** Phenolic compounds profile (mg kg^−1^) in the skin of ‘Bordô’ grapes under different training systems and rootstocks.

Compounds *	Trellis	Rootstock		CV (%)
		‘IAC 766 Campinas’	‘106-8 Mgt’	
Anthocyanins				
Malvidin-3,5-diglycoside	Low	133.51 ± 3.47 bA	134.06 ± 2.08 bA	1.73
	High	150.18 ± 2.95 aB	157.33 ± 2.02 aA	
Delphinidin-3-O-glucoside	Low	18.02 ± 0.15 bA	16.71 ± 0.001 bB	0.55
	High	20.24 ± 0.17 aA	18.54 ± 0.02 aB	
Cyanidin-3-O-glucoside	Low	1.65 ± 0.02 bA	1.59 ± 0.01 bA	2.27
	High	2.21 ± 0.07 aA	1.85 ± 0.04 aB	
Peonidin-3-O-glucoside	Low	1.28 ± 0.03 bA	1.01 ± 0.01 bB	1.10
	High	1.37 ± 0.01 aA	1.14 ± 0.001 aB	
Malvidin-3-O-glucoside	Low	7.74 ± 0.05 aA	6.56 ± 0.01 bB	1.05
	High	7.41 ± 0.13 bB	7.90 ± 0.06 aA	
Flavonol				
Rutin	Low	8.67 ± 0.16 bA	6.76 ± 0.10 bB	1.41
	High	9.59 ± 0.13 aA	9.64 ± 0.11 aA	
Phenolic acids				
Caffeic acid	Low	2.52 ± 0.05 bA	2.50 ± 0.04 bA	2.19
	High	4.29 ± 0.07 aB	4.51 ± 0.09 aA	
Chlorogenic acid	Low	3.39 ± 0.05 bA	3.13 ± 0.02 bB	1.09
	High	4.17 ± 0.02 aA	3.93 ± 0.04 aB	
*p*-coumaric acid	Low	0.44 ± 0.001 aA	0.44 ± 0.001 bA	0.65
	High	0.44 ± 0.001 aB	0.45 ± 0.001 aA	
*t*-ferulic acid	Low	1.19 ± 0.001 bA	1.18 ± 0.001 aB	0.22
	High	1.20 ± 0.001 aA	1.17 ± 0.001 bB	
Total	Low	178.42 ± 3.99 bA	173.96 ± 2.28 bA	1.52
	High	201.11 ± 3.40 aA	206.48 ± 2.38 aA	

Means followed by the same lowercase letter in the column and uppercase letter in the row do not differ from each other by the Tukey test at a 5% probability level. * Compounds in mg kg^−1^ of skins. CV: Coefficient of Variation; dig.: diglycoside; g.: glycoside.

**Table 4:** Total flavonoids (TF), total phenolic compounds (TPC), and antioxidant activity (DPPH and FRAP) in the skins, as well as total flavonoids (TF) and antioxidant activity (DPPH), in the pulp of ‘Isabel’ grapes under different training systems and rootstocks.

Variable	Trellis		Rootstock		CV (%)
	Low	High	‘IAC 766 Campinas’	‘106-8 Mgt’	
Skin					
TF (mg 100 g^−1^)	32.41 ± 4.56 a	31.99 ± 4.56 a	35.21 ± 3.79 a	29.19 ± 2.58 b	9.93
DPPH (mmol g^−1^)	5.19 ± 0.80 b	7.05 ± 1.39 a	5.85 ± 1.42 a	6.39 ± 1.54 a	19.31
FRAP (mmol Fe kg^−1^)	85.78 ± 2.52 b	94.65 ± 1.79 a	89.80 ± 5.80 a	90.63 ± 4.53 a	2.52
TPC (mg 100 g^−1^)	293.35 ± 17.46 b	379.58 ± 36.32 a	352.95 ± 59.51 a	319.98 ± 41.36 b	6.49
Pulp					
TF (mg 100 g^−1^)	0.42 ± 0.02 b	0.53 ± 0.08 a	0.44 ± 0.07 b	0.51 ± 0.08 a	8.90
DPPH (mmol g^−1^)	0.53 ± 0.13 a	0.46 ± 0.16 a	0.53 ± 0.09 a	0.46 ± 0.16 a	23.87

Means followed by the same lowercase letter in the row, within the same factor, do not differ from each other according to the Tukey test at a 5% probability level. CV: Coefficient of Variation.

**Table 5:** Total monomeric anthocyanins (TMA) in the skins, total phenolic compounds (TPC), total monomeric anthocyanins (TMA), and antioxidant activity (FRAP) in the pulp of ‘Isabel’ grapes under different training systems and rootstocks.

Variable	Trellis	Rootstock		CV (%)
		‘IAC 766 Campinas’	‘106-8 Mgt’	
Skin				
TMA (mg 100 g^−1^)	Low	96.49 ± 2.56 bB	122.17 ± 0.78 bA	1.30
	High	166.02 ± 1.03 aA	127.86 ± 1.72 aB	
Pulp				
TPC (mg 100 g^−1^)	Low	16.68 ± 1.26 bA	16.89 ± 0.85 aA	9.18
	High	20.19 ± 1.82 aA	16.46 ± 2.18 aB	
TMA (mg 100 g^−1^)	Low	0.23 ± 0.03 bA	0.18 ± 0.03 aB	17.65
	High	0.38 ± 0.07 aA	0.18 ± 0.01 aB	
FRAP (mmol Fe kg^−1^)	Low	5.77 ± 0.60 bA	4.89 ± 0.66 aB	9.82
	High	8.94 ± 0.64 aA	5.14 ± 0.53 aB	

Means followed by the same lowercase letter in the column and uppercase letter in the row do not differ from each other according to the Tukey test at a 5% probability level. CV: Coefficient of Variation.

**Table 6:** Anthocyanins, flavonols, and phenolic acids (mg kg^−1^) in the skins of ‘Isabel’ grapes under different training systems and rootstocks.

Compounds *	Trellis	Rootstock		CV (%)
		‘IAC 766 Campinas’	‘106-8 Mgt’	
Anthocyanins				
Malvidin-3,5-diglycoside	Low	9.43 ± 0.08 bA	5.88 ± 0.15 bB	1.35
	High	12.32 ± 0.19 aA	8.49 ± 0.05 aB	
Delphinidin-3-O-glucoside	Low	14.97 ± 0.02 bA	14.36 ± 0.07 bB	0.28
	High	15.83 ± 0.04 aA	14.68 ± 0.001 aB	
Cyanidin-3-O-glucoside	Low	1.64 ± 0.01 bA	0.80 ± 0.03 aB	1.70
	High	1.72 ± 0.04 aA	0.74 ± 0.01 bB	
Peonidin-3-O-glucoside	Low	9.61 ± 0.01 bA	7.07 ± 0.23 bB	1.33
	High	10.46 ± 0.11 aA	8.09 ± 0.02 aB	
Malvidin-3-O-glucoside	Low	19.72 ± 0.08 bA	12.95 ± 0.32 bB	0.79
	High	22.09 ± 0.30 aA	20.34 ± 0.03 aB	
Flavonol				
Rutin	Low	8.58 ± 0.01 bB	9.89 ± 0.19 aA	1.03
	High	10.24 ± 0.11 aA	9.00 ± 0.01 bB	
Phenolic acids				
3-hydroxytyrosol acid	Low	0.02 ± 0.001 bA	0.03 ± 0.01 aA	11.77
	High	0.04 ± 0.01 aA	0.03 ± 0.01 aA	
Caffeic acid	Low	0.72 ± 0.01 aB	0.80 ± 0.01 aA	0.69
	High	0.67 ± 0.01 bA	0.68 ± 0.01 bA	
Chlorogenic acid	Low	2.39 ± 0.01 bA	2.40 ± 0.01 aB	0.33
	High	2.42 ± 0.01 aA	2.38 ± 0.01 bB	
*p*-coumaric acid	Low	0.42 ± 0.001 aA	0.40 ± 0.001 aB	1.35
	High	0.40 ± 0.001 bA	0.40 ± 0.001 aA	
*t*-cinnamic acid	Low	1.16 ± 0.001 bA	1.14 ± 0.001 bB	0.48
	High	1.17 ± 0.001 aA	1.16 ± 0.001 aA	
*t*-ferulic acid	Low	1.18 ± 0.001 aA	1.16 ± 0.001 bB	0.25
	High	1.16 ± 0.01 bB	1.17 ± 0.001 aA	
Total	Low	68.68 ± 0.01 bA	55.74 ± 1.00 bB	0.72
	High	77.31 ± 0.70 aA	66.01 ± 0.06 aB	

Means followed by the same lowercase letter in the column and uppercase letter in the row do not differ from each other according to the Tukey test at a 5% probability level. * Compounds in mg kg^−1^ of skins. CV: Coefficient of Variation; dig.: diglycoside; g.: glycoside.

**Table 7:** Total flavonoids (TF), total phenolic compounds (TFC), total monomeric anthocyanins (TMA), and antioxidant activity (DPPH) of the skin and total monomeric anthocyanins (TMA) of the pulp of the IAC 138-22 ‘Máximo’ grape in different training systems and rootstocks.

Variable	Trellis	Rootstock		CV (%)
		‘IAC 766 Campinas’	‘106-8 Mgt’	
Skin				
TF (mg 100 g^−1^)	Low	116.12 ± 18.33 aA	95.07 ± 5.99 bA	15.31
	High	116.06 ± 11.48 aA	136.23 ± 27.49 aA	
TPC (mg 100 g^−1^)	Low	930.89 ± 3.64 bA	904.34 ± 1.65 bB	1.07
	High	1059.67 ± 18.30 aA	986.80 ± 8.89 aB	
TMA (mg 100 g^−1^)	Low	653.17 ± 38.79 aA	435.78 ± 23.45 bB	9.35
	High	646.10 ± 35.12 aA	605.89 ± 30.56 aA	
DPPH (mmol g^−1^)	Low	14.27 ± 0.69 bB	22.44 ± 2.00 bA	8.84
	High	24.49 ± 3.11 aA	25.58 ± 0.74 aA	
Pulp				
TMA (mg 100 g^−1^)	Low	1.29 ± 0.14 bB	2.66 ± 0.27 aA	14.69
	High	2.06 ± 0.54 aB	2.64 ± 0.15 aA	

Means followed by the same lowercase letter in the column and uppercase letter in the row do not differ from each other according to the Tukey test at a 5% probability level. CV: Coefficient of Variation.

**Table 8:** Antioxidant activity (FRAP) of the skin and total flavonoids (TF), total phenolic compounds (TPC), and antioxidant activity (DPPH and FRAP) of the pulp of the IAC 138-22 ‘Máximo’ grape in different training systems and rootstocks.

Variable	Trellis		Rootstock		CV (%)
	Low	High	‘IAC 766 Campinas’	‘106-8 Mgt’	
Skin					
FRAP (Mmol Fe kg^−1^)	280.80 ± 25.85 a	281.02 ± 27.15 a	257.56 ± 11.89 b	304.26 ± 4.26 a	3.35
Pulp					
TF (mg 100 g^−1^)	1.01 ± 0.22 b	1.31 ± 0.44 a	1.36 ± 0.43 a	0.95 ± 0.08 b	22.92
TPC (mg 100 g^−1^)	16.96 ± 3.33 a	12.78 ± 2.07 b	13.68 ± 3.79 a	16.06 ± 2.73 a	17.66
DPPH (mmol g^−1^)	0.30 ± 0.13 a	0.25 ± 0.13 b	0.39 ± 0.04 a	0.16 ± 1.15 b	10.79
FRAP (mmol Fe kg^−1^)	8.55 ± 3.99 b	9.70 ± 3.99 a	12.89 ± 0.04 a	5.35 ± 0.63 b	8.08

Means followed by the same lowercase letter in the row, within the same factor, do not differ from each other according to the Tukey test at a 5% probability level. CV: Coefficient of Variation.

**Table 9:** Phenolic compounds (mg kg^−1^) of the skin of the IAC 138-22 ‘Máximo’ grape in different training systems and rootstocks.

Compounds *	Trellis	Rootstock		CV (%)
		‘IAC 766 Campinas’	‘106-8 Mgt’	
Anthocyanins				
Malvidin-3.5-diglycoside	Low	108.73 ± 0.98 aB	152.45 ± 0.57 aA	0.44
	High	106.10 ± 0.89 bB	146.22 ± 0.26 bA	
Delphinidin-3-O-glucoside	Low	23.60 ± 0.18 bB	34.61 ± 0.15 aA	0.70
	High	27.13 ± 0.26 aB	30.22 ± 0.08 bA	
Cyanidin-3-O-glucoside	Low	1.50 ± 0.20 aB	2.32 ± 0.05 aA	5.61
	High	1.44 ± 0.02 aB	1.96 ± 0.02 bA	
Peonidin-3-O-glucoside	Low	2.37 ± 0.03 bB	3.51 ± 0.01 aA	1.09
	High	3.18 ± 0.04 aB	3.25 ± 0.03 bA	
Malvidin-3-O-glucoside	Low	28.79 ± 0.14 bB	43.91 ± 0.28 aA	0.46
	High	33.41 ± 0.15 aB	42.68 ± 0.12 bA	
Flavonol				
Rutin	Low	16.37 ± 0.20 bB	29.07 ± 0.29 aA	0.35
	High	18.11 ± 0.10 aB	28.32 ± 0.18 bA	
Phenolic acids				
3-hydroxytyrosol acid	Low	0.09 ± 0.01 bA	0.09 ± 0.01 bA	7.31
	High	0.04 ± 0.001 aB	0.13 ± 0.001 aA	
Caffeic acid	Low	1.05 ± 0.001 bB	1.38 ± 0.001 aA	0.28
	High	1.46 ± 0.01 aA	0.91 ± 0.001 bB	
Chlorogenic acid	Low	2.70 ± 0.04 bB	3.30 ± 0.04 aA	1.29
	High	3.43 ± 0.04 aA	2.90 ± 0.03 bB	
*p*-coumaric acid	Low	0.45 ± 0.001 bB	0.48 ± 0.001 aA	0.71
	High	0.47 ± 0.001 aA	0.46 ± 0.001 bB	
*t*-cinnamic acid	Low	1.21 ± 0.01 bB	1.29 ± 0.001 aA	0.38
	High	1.22 ± 0.001 aB	1.25 ± 0.01 bA	
*t*-ferulic acid	Low	1.20 ± 0.01 aB	1.23 ± 0.01 aA	0.31
	High	1.20 ± 0.001 aB	1.22 ± 0.01 bA	
Flavan-3-ol				
Catechin	Low	1.15 ± 0.01 bB	2.42 ± 0.33 aA	8.90
	High	2.76 ± 0.06 aA	1.63 ± 0.02 bB	
Total	Low	186.78 ± 1.07 bB	272.29 ± 1.40 aA	0.30
	High	195.94 ± 1.40 aB	258.18 ± 0.05 bA	

Means followed by the same lowercase letter in the column and uppercase letter in the row do not differ from each other according to the Tukey test at a 5% probability level. * Compounds in mg kg^−1^ of skins. CV: Coefficient of Variation; dig.: diglycoside; g.: glycoside.

**Table 10:** Total flavonoids (TF) and antioxidant activity (DPPH) of the skin and antioxidant activity (DPPH) of the pulp of the ‘BRS Violeta’ grape in different training systems and rootstocks.

Variable	Trellis		Rootstock		CV (%)
	Low	High	‘IAC 766 Campinas’	‘106-8 Mgt’	
Skin					
TF (mg 100 g^−1^)	119.02 ± 31.10 b	167.97 ± 17.23 a	130.57 ± 34.75 b	156.42 ± 32.31 a	15.21
DPPH (mmol g^−1^)	40.99 ± 7.26 a	39.89 ± 4.06 a	43.84 ± 3.11 a	37.04 ± 5.82 b	12.08
Pulp					
DPPH (Mmol g^−1^)	0.60 ± 0.14 b	0.72 ± 0.04 a	0.63 ± 0.14 a	0.68 ± 0.08 a	15.66

Means followed by the same lowercase letter in the row, within the same factor, do not differ from each other according to the Tukey test at a 5% probability level. CV: Coefficient of Variation.

**Table 11:** Total phenolic compounds (TPC), total monomeric anthocyanins (TMA), and antioxidant activity (FRAP) of the skin, and total flavonoids (TF), total phenolic compounds (TPC), total monomeric anthocyanins (TMA), and antioxidant activity (FRAP) of the pulp of the ‘BRS Violeta’ grape in different training systems and rootstocks.

Variable	Trellis	Rootstock		CV (%)
		‘IAC 766 Campinas’	‘106-8 Mgt’	
		Skin		
TPC (mg 100 g^−1^)	Low	1080.44 ± 8.80 bB	1518.77 ± 23.45 bA	1.81
	High	1505.97 ± 40.27 aB	1600.34 ± 20.77 aA	
TMA (mg 100 g^−1^)	Low	815.67 ± 13.85 bB	1330.77 ± 49.62 aA	6.88
	High	1296.57 ± 47.90 aA	1410.81 ± 46.89 aA	
FRAP (Mmol Fe kg^−1^)	Low	513.80 ± 10.63 bB	637.40 ± 6.49 aA	1.36
	High	569.74 ± 3.32 aB	639.87 ± 9.51 aA	
Pulp				
TF (mg 100 g^−1^)	Low	0.85 ± 0.15 bA	0.94 ± 0.04 aA	9.51
	High	1.00 ± 0.06 aA	0.86 ± 0.07 aB	
TPC (mg 100 g^−1^)	Low	41.29 ± 13.94 aA	24.17 ± 2.04 aB	24.98
	High	26.36 ± 3.21 bA	24.30 ± 1.24 aA	
TMA (mg 100 g^−1^)	Low	1.28 ± 0.31 bA	0.91 ± 0.04 aB	14.82
	High	1.85 ± 0.13 aA	0.51 ± 0.02 bB	
FRAP (Mmol Fe kg^−1^)	Low	13.28 ± 0.69 bB	16.57 ± 0.82 bA	4.41
	High	18.31 ± 1.01 aA	19.12 ± 0.18 aA	

Means followed by the same lowercase letter in the column and uppercase letter in the row do not differ from each other according to the Tukey test at a 5% probability level. CV: Coefficient of Variation.

**Table 12:** Phenolic compounds (mg kg^−1^) of the skin of the ‘BRS Violeta’ grape in different training systems and rootstocks.

Compounds *	Trellis	Rootstock		CV (%)
		‘IAC 766 Campinas’	‘106-8 Mgt’	
Anthocyanins				
Malvidin-3,5-diglycoside	Low	539.06 ± 14.69 bA	93.38 ± 3.82 bB	2.08
	High	665.00 ± 1.47 aA	117.81 ± 0.28 aB	
Delphinidin-3-O-glucoside	Low	218.52 ± 5.32 bA	177.01 ± 9.64 bB	3.68
	High	256.48 ± 0.70 aA	198.85 ± 17.90 aB	
Cyanidin-3-O-glucoside	Low	194.72 ± 3.44 bA	164.24 ± 11.96 bB	3.93
	High	246.87 ± 1.55 aA	199.44 ± 6.59 aB	
Peonidin-3-O-glucoside	Low	26.04 ± 0.64 bA	0.58 ± 0.01 aB	2.39
	High	37.67 ± 0.27 aA	0.60 ± 0.001 aB	
Malvidin-3-O-glucoside	Low	2.01 ± 0.01 bB	2.30 ± 0.03 bA	0.80
	High	2.05 ± 0.001 aB	2.43 ± 0.04 aA	
Flavonol				
Rutin	Low	10.91 ± 0.25 bA	10.01 ± 0.10 bB	0.96
	High	13.26 ± 0.03 aB	15.31 ± 0.03 aA	
Phenolic acids				
3-hydroxytyrosol acid	Low	0.21 ± 0.02 bB	0.26 ± 0.001 aA	4.45
	High	0.26 ± 0.01 aA	0.27 ± 0.01 aA	
Caffeic acid	Low	1.92 ± 0.04 aA	1.84 ± 0.06 bB	1.68
	High	1.21 ± 0.001 bB	2.17 ± 0.001 aA	
Chlorogenic acid	Low	4.34 ± 0.04 aA	3.72 ± 0.05 bB	0.97
	High	3.49 ± 0.03 bB	4.33 ± 0.001 aA	
*p*-coumaric acid	Low	0.64 ± 0.001 bA	0.59 ± 0.001 bB	0.45
	High	0.66 ± 0.001 aB	0.69 ± 0.001 aA	
*t*-innamic acid	Low	1.22 ± 0.001 bA	1.19 ± 0.01 aB	0.57
	High	1.24 0.001 aA	1.17 ± 0.001 bB	
*t*-ferulic acid	Low	1.16 ± 0.001 bA	1.17 ± 0.01 aA	0.55
	High	1.19 ± 0.001 aA	1.17 0.001 aB	
Total	Low	804.62 ± 20.96 bA	290.63 ± 13.66 bB	1.72
	High	981.00 ± 2.51 aA	343.37 ± 17.60 aB	

Means followed by the same lowercase letter in the column and uppercase letter in the row do not differ from each other according to the Tukey test at a 5% probability level. * Compounds in mg kg^−1^ of skins. CV: Coefficient of Variation; dig.: diglycoside; g.: glycoside.

## Data Availability

The original contributions presented in the study are included in the article material; further inquiries can be directed to the corresponding author.
